# Secret hiding places in the eye and beyond: what about after SARS-CoV-2 infection?

**DOI:** 10.1007/s00417-021-05323-9

**Published:** 2021-08-03

**Authors:** Thomas C. Erren, Philip Lewis

**Affiliations:** Institute and Policlinic for Occupational Medicine, Environmental Medicine and Prevention Research, University of Cologne, Kerpener Str. 61, 50938 Cologne, Germany

Dear Editor:

In a prospective study, Rokohl and colleagues looked into the possibility that SARS-CoV-2 may hide in the eyes of pre-screened asymptomatic patients in a tertiary eye care center [1]. The authors explored this possibility in 1145 patients of whom none had any real-time reverse transcriptase-polymerase chain reaction (RT-PCR)–confirmed virus-positive result from nasopharyngeal swabs, suggesting that all study individuals were *uninfected.* Regarding conjunctival swabs, all tested eyes yielded virus-negative results. Clearly, this is important empirical information in regard to infection risks for ophthalmologists and medical staff in tertiary eye care centers, including the handling of human corneal donor tissue — always provided that patients or donors are *not infected*.

We compliment the authors for taking this initiative and call for research regarding hiding places of SARS-CoV-2 to be expanded: What about *infected* individuals, viz., COVID-19 patients, survivors, or asymptomatic carriers, and virus material persistence in immune-privileged sites of the eye and elsewhere?

To exemplify why “secret” hiding sites in *infected* individuals may be(come) of interest, in 1967 a virus reservoir in sperm was proposed in the course of discovering the Marburg virus (MV) [2]. Careful observations had suggested that several diseased men’s testes were also infected and abstaining from sex until further notice was recommended after hospital dismissal. Yet, after one MV patient left hospital and had intercourse with his wife, she developed symptoms and signs of Marburg disease. From the overall evidence, it was concluded that the infection occurred via virus-containing ejaculate. In other words, an until then unappreciated (described as “unusual” [2]) spermatogeneous infection route from an MV reservoir which existed for almost 4 months was suggested. Regarding another filovirus, sexual transmission from a male survivor of Ebola virus disease [EVD] to his female partner was suspected in 2015 [3]. That Ebola virus RNA can persist many months after manifestation of disease [4] — in semen from﻿﻿ an EVD survivor with pre-existing HIV infection it was detected 565 days after acute disease [5] — conveys that understanding virus clearance and remaining virus “potential” from immune-privileged sites/organs is important for individuals and possibly public health [6].

Regarding SARS-CoV-2, investigating virus materials in semen of COVID-19 cases in acute and recovery phases yielded ambiguous results. The question of whether virus material may persist in the privileged immunity site of the testes remains open [7]. In contrast, regarding SARS-CoV-2 in the eye, Rokohl and colleagues pointed out that the virus was unambiguously detected in ocular tissues, tears, and conjunctival secretions of COVID-19 patients [1]. The key question of whether long(er)-term SARS-CoV-2 persistence in immune privileged sites such as in the eyes﻿﻿ [8] of COVID-19 survivors or asymptomatic carriers may hold long(er)-term risks is empirically open and should be explored with targeted research [9, 10].

## Data Availability

Not applicable.
